# Variation of the steroid profile in relation to training in women and its importance for anti-doping testing

**DOI:** 10.3389/fphys.2026.1733515

**Published:** 2026-03-04

**Authors:** A. Andersson, L. Ekström, O. Salamin, R. Nicoli, A. L. Hirschberg, E. Eklund

**Affiliations:** 1 Department of Laboratory Medicine, Karolinska Institutet, Stockholm, Sweden; 2 Medical Unit of Clinical Pharmacology, Karolinska University Hospital, Stockholm, Sweden; 3 Swiss Laboratory for Doping Analyses, University Center of Legal Medicine, Lausanne, Switzerland; 4 Lausanne University Hospital and University of Lausanne, Lausanne, Switzerland; 5 Department of Women’s and Children’s Health, Karolinska Institutet, Stockholm, Sweden; 6 Department of Gynecology and Reproductive Medicine, Karolinska University Hospital, Stockholm, Sweden

**Keywords:** androgens, anti-doping, dried blood spot (DBS), exercise, female athletes, testosterone, urinary steroids

## Abstract

**Introduction:**

The urinary steroid module of the Athlete Biological Passport (ABP), monitoring biomarkers over time is limited in female athletes. A serum steroid module has been implemented, including testosterone (T), androstenedione (A4) and the T/A4 ratio, being more stable regarding hormonal fluctuations in women. Acute training may increase serum T and decrease the urinary excretion of androgens. Moreover, the urinary levels of ABP metabolites have been shown to be lower in female athletes compared to sedentary controls. One hypothesis is elimination of some of the androgens via sweat. Therefore, it is of interest to study the urinary and circulatory steroids in relation to training and sweat production.

**Material and methods:**

30 healthy female athletes and 26 untrained BMI-matched controls were included. The athlete’s urine and fluid intake was collected over 48 h during a rest- and a training day, and the controls for 24 sedentary hours. Estimated sweat loss was calculated. For the athletes, dried blood spots (DBS) were collected at rest, before and after training and the day after training. Urine was analyzed for the urinary steroid profile by gas chromatography–tandem mass spectrometry and DBS for T and A4 by liquid chromatography–tandem mass spectrometry.

**Results:**

Sweat production was elevated in athletes during the training day versus the rest day, but there were no differences compared to the controls. No significant intra-individual variation (CV %) in urinary steroid profiles was observed; however, controls excreted higher absolute levels of urinary A and 5αAdiol. In DBS, T remained stable whereas a minor increase in A4 was noted in samples taken the day after training. For the T/A4 ratio changes were observed in samples taken after exercise only.

**Conclusion:**

T and A4 in DBS were not affected by acute training. As DBS sample time differed during the day the minor changes in A4 and the T/A4 ratio may be due to diurnal variation and not training dependent effects. In urine certain urinary steroids were lower in the female athletes compared to controls. These results may be of interest when interpreting results of the ABP.

## 1 Introduction

The Athlete Biological Passport (ABP) was adopted by the World Anti-Doping Agency (WADA) to longitudinally monitor steroid biomarkers (WADA, 2021). While the urinary module of the ABP has proven effective for male athletes, its utility in female athletes is more limited. This is primarily due to lower baseline androgen concentrations, greater hormonal fluctuations associated with the menstrual cycle, and the use of hormonal contraceptives (HC). These factors introduce significant variability to the steroid profile, thereby widening the tolerance thresholds in the ABP and reducing its sensitivity (Elings Knutsson et al., 2021; Schulze et al., 2021; Ekström et al., 2023).

We have recently demonstrated that female Olympic athletes exhibit lower urinary androgen levels compared to sedentary controls even though serum androgen levels were comparable. Furthermore, the urinary androgen levels negatively correlated with training volume (Eklund et al., 2021). Similar findings were reported by Timon et al., showing lower urine steroid profile values in trained male cyclists than sedentary controls (Timon et al., 2008a). Since a possible eliminations path of androgens may occur via sweat (Thieme et al., 2003), we hypothesized that the increased sweating during intense training over time, may contribute to the reduction in urinary androgen concentrations in highly trained athletes.

Initially, the ABP focused solely on the urinary steroid profile, but it has since been expanded to include serum testosterone (T) (WADA, 2023). Androstenedione (A4) was also introduced to form the ratio T/A4 which is considered more stable and sensitive than T alone and less affected by menstrual cycle and hormonal contraceptives (Salamin et al., 2022b; Knutsson et al., 2023). While various confounding factors have been extensively studied for the urinary model, they remain less explored in the serum-based model. The effect of training/exercise on T and A4 levels in women is not clear. Evidence supports an increase in T after acute exercise although this normalizes to baseline or lower within hours (Enea et al., 2011; Hackney and Willett, 2020). Long-term exercise studies have shown more variability, most likely attributed to different study designs (Enea et al., 2011; Swain et al., 2022).

Dried blood spots (DBS) collected via capillary sampling have been implemented as a complementary matrix in anti-doping testing, mainly in situations where venous blood collection is challenging (Thevis et al., 2016). Even though routine T and A4 is analyzed in serum today, DBS may be employed for blood steroids profiling provided that all comparisons are conducted within the same matrix (Salamin et al., 2021; Salamin et al., 2022a). Studies have shown that analysis of T and A4 in DBS is reliable to detect micro-dosing of T in healthy females (Desai et al., 2024). In light of its minimally invasive nature and suitability for remote or home-based sampling, DBS was selected as the preferred matrix for this study.

The aim of the present study was to investigate the urinary steroid profile and estimated sweat production during a non-training day and a training day in female athletes training on average 2–3 h per day compared to controls training less than 2 h per week. In addition, for the athlete’s group, we aimed to study the acute effect of exercise on T and A4 concentrations when measured in DBS.

## 2 Materials and methods

### 2.1 Study population and data collection

In this study, 30 healthy female athletes exercising on average 2–3 h per day and 26 untrained BMI-matched control women exercising maximum 2 h per week, were included. Participants were recruited via advertisements (www.accidi.se). Female athletes were eligible for inclusion if they were healthy, premenopausal, nonpregnant, over 18 years of age, trained on average a minimum of 2–3 h per day, and willing to refrain from training for two consecutive days. Control participants met the following inclusion criteria: over 18 years of age, healthy, premenopausal, not pregnant, BMI-matched to the athletes, and training ≤2 h per week.

**FIGURE 1:**
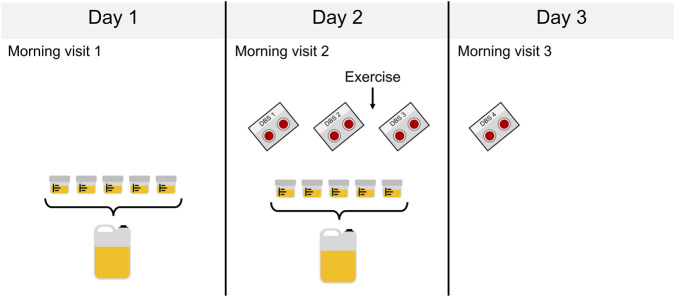
The study sample collection scheme over three consecutive days. For the athletes spot urine samples were collected on day 1 (non-training day) and 2 (training day), and for the controls on day 1. 2 mL of each spot urine sample was aliquoted for GC-MS/MS analysis after which all urine was pooled by day. For the athletes dried blood spots (DBS) were collected. DBS 1 and 4 was collected in the morning at the clinic: DBS 1 (baseline sample) at day 2 study visit 2 and at day 3 study visit 3. DBS 2 (pre-training sample) was collected by home-base sampling at day 2 immediately prior to exercise and DBS 3 (post-training sample) on the same day immediately after exercise.

Recruitment was initiated in November 2023 and completed in January 2025. Participants were examined at the Women’s Health Research Unit, Karolinska University Hospital, with all visits occurring between 08:00 and 10:00 am. The athletes completed the study protocol over three consecutive days, and controls over two consecutive days. For sampling regiment, see Figure 1. Physical activity was not allowed the day before study visit 1 or the first day of the study (time between study visit 1 and 2).

All participants filled out a general health questionnaire including general health parameters, HC use, pregnancy, and training hours per week. Blood pressure, heart rate, height, and weight were measured at all visits.

For the athletes, 48 h urine sampling and fluid intake/spot urine diary (24 h during a non-training period and 24 h during a training period) were collected. For the controls, urine samples and fluid intake/spot urine diary were collected during a 24 h non-training period. During the study period, participants collected the full urine volume from every micturition (spot urine samples). Two ml of each spot urine sample was then withdrawn for analyses, and then the remaining urine was pooled by day for analyses (Figure 1).

In athletes, DBS samples were collected on four occasions (see Figure 1) by capillary blood sampling using a single-use lancet (Accu-Chek Safe-T-Pro plus) and Capitainer collection divide (Capitainer®B50, 2 × 50 µL). DBS sample 1 (baseline) and 4 (follow-up) were collected in the morning (between 08.00 and 10.00 a.m.) during study visit 2 (day 2) and 3 (day 3) and DBS 2 (pre-training) and 3 (post-training) by home-based testing just before and after the study participants performed their regular training sessions on day 2. The athletes regular training sessions consisted mainly of endurance exercise although some partook in only strength training or a combination of both.

Sweat production (over 24 h for the controls and two 24 h periods (non-training and training) for the athletes) was estimated as described by Cheuvront et al. (2002) with slight alternations, accordingly: (weight visit 1 – weight visit 2) + fluid intake day 1- urine volume day 1 and for the athletes also for the training period: (weight visit 2- weight visit 3) + fluid intake day 2 –urine volume day 2.

The study was conducted according to the Declaration of Helsinki and was approved by the Regional Ethics Committee, Stockholm (EPN 2023-00161–01). Informed consent was obtained from all participants. Research participants could at any time cancel their participation in the study.

### 2.2 Urine analyses

All parameters included in the urinary steroidal ABP module - T, epitestosterone (E), androsterone (A), etiocholanolone (Etio), 5α-androstanediol (5αAdiol) and 5β-androstanediol (5βAdiol) - were determined using a WADA-accredited gas chromatography mass spectrometry (GC-MS/MS) method at the Karolinska University Hospital, Huddinge. The applied method was a modification of what has previously been described by Mullen et al. (2017) and Bohlin et al. (2024). In short: internal standard, phosphate buffer (pH 6.5) and β-glucuronidase from *E. coli* was added to 2 mL sample. The mixture was incubated for 60 min at 50 °C. Once cooled, the sample was extracted using potassium carbonate and methyl tert-butyl ether which was dried using sodium sulfate. After centrifugation the water phase was frozen, and the ether decanted into a fresh tube. The sample was reconstituted in acetone after evaporation and transferred to injection vials. The dried sample was derivatized using 40 μL derivatization reagent for 30 min at 80 °C. The sample was then analyzed on an Agilent 7000C Triple Quadrupole GC/MS (Agilent Technologies, Santa Clara, CA, United States of America).

Specific gravity (SG) was measured with an ATAGO UG-1 digital refractometer (Atago Co., Ltd., Tokyo, Japan) to adjust for the dilution of the urine. All urinary parameter concentrations were normalized using specific gravity: normalized concentration (ng/mL) = measured concentration (ng/mL) × 0.02 × (SG – 1)^−1^ (WADA, 2021).

### 2.3 DBS analyses

A single 50 µL DBS was used for the extraction and quantification of T and A4. Extraction was performed in two sequential steps: first with 500 µL of methanol:isopropanol (1:1, v/v) containing the internal standards T-d3 and A4-d7, followed by 500 µL of tert-butyl methyl ether (TBME). After each solvent addition, samples were incubated on a thermoshaker for 25 min at 25 °C. Combined extracts were evaporated to dryness and reconstituted in 100 µL of methanol:water (50:50, v/v).

Chromatographic separation was achieved on an Acquity I-Class LC system (Waters) coupled to a Xevo TQ-S triple quadrupole mass spectrometer (Waters). The system was operated under the gradient, source, and MRM conditions previously described (Langer et al., 2022). Mobile phase A consisted of 5 mM ammonium formate in water and mobile phase B of 5 mM ammonium formate in methanol. An Ethylene Bridged Hybrid (BEH) C18 column (100 × 2.1 mm, 1.7 µm; Waters) was maintained at 60 °C, and the injection volume was 10 µL.

Quantification was performed using a 5-point calibration curve prepared as described in (Salamin et al., 2021) with Capitainer®B50 cards. Linear calibration models were generated for each analyte by plotting the peak area ratio of the analyte to its corresponding internal standard against concentration, applying 1/x weighted least-squares regression. Data acquisition and processing were performed using MassLynx v4.2 and TargetLynx (Waters).

### 2.4 Statistical analysis

Continuous data was presented as mean ± SD when normally distributed or as median and interquartile range (25th–75th percentile) otherwise.

For comparison of age, baseline height, weight, BMI, heart rate and blood pressure and amount of training between athletes and controls the student´s t-test was applied. For comparison of HC between groups the Pearson Chi-square test was applied.

The total urinary concentrations and estimated sweat production showed non-parametric distribution (Shapiro-Wilk test), except Etio which was normally distributed. Subsequently, for comparison of urinary steroids and sweat production in athlete’s day 1 and day 2 Wilcoxon matched pair test was performed, whereas for Etio a paired t-test was done. Comparing sweat production and urinary concentrations between athlete groups and controls, the Kruskal–Wallis test was used, followed by Dunn’s multiple comparison test.

T and T/A4 were not normally distributed and were therefore log-transformed before repeated measurement ANOVA (RM-ANOVA). Participants 5, 6, and 13 were excluded from RM-ANOVA analyses due to incomplete datasets. Participants 8 and participant 18 were excluded because each presented a single outlier. Sphericity was verified using Mauchly’s Test of Sphericity. A Bonferroni correction was applied to the post-hoc analysis.

Descriptive statistics and figures were produced with GraphPad Prism (v10) and R Statistical Software (R Core Team, 2023) with ggplot2 package (Wickham H ggplot2, 2016). RM-ANOVA was analyzed with the rstatix package (Kassambara, 2023). A significance level of 0.05 was applied throughout the analyses.

## 3 Results

### 3.1 Baseline characteristics

In Table 1, baseline characteristics of the study groups are presented. There was no significant difference in age, height, weight, BMI, blood pressure or HC use between the female athletes and the controls. The controls had significantly higher heart rate compared to the athletes (p = 0.047). As expected, the athletes had significantly higher training hours per week compared to the controls (p < 0.001).

**TABLE 1:** General baseline characteristics, heart rate, blood pressure, HC use and amount of training.

Parameter	Athletes	Controls
n	30	26
Age	27.4 ± 5.1	26.3 ± 5.4
Height (m)	1.67 ± 0.08	1.68 ± 0.08
Weight (kg)	59.4 ± 7.6	62.7 ± 9.0
BMI	21.3 ± 2.5	22.2 ± 2.8
Heart rate (bpm)	68.0 ± 11.3	74.8 ± 13.1 *
BP systolic (mm Hg)	113.8 ± 9.2	113.0 ± 8.8
BP diastolic (mm Hg)	71.6 ± 7.5	74.0 ± 6.9
HC use, n (%)	12 (40%)	9 (35%)
Amount of training (h/w)	15.8 ± 5.7	1.3 ± 0.7 ***

Values presented as mean ± SD., for comparison of age, height, weight; BMI, heart rate and blood pressure and amount of training between groups the student´s t-test was applied. For comparison of HC, between groups the Pearson Chi-square test was applied.

BMI, body mass index; BP, blood pressure; bpm, beats per minute; HC, hormonal contraceptive; kg, kilograms; m, meters; mmHg, millimetres of mercury; h/w, hours per week; n, number.

*p < 0.05, **p < 0.01, ***p < 0.001.

### 3.2 Estimated sweat production

In the athletes, estimated sweat production was significantly higher during day 2 (training period) compared to day 1 (non-training period), p = 0.025 (Table 2). When comparing estimated sweat production between the athletes and the controls, no significant difference was observed. In the athletes, no significant correlations were found between estimated sweat production day 1 and total urinary steroids day 1 nor between sweat production day 2 and urinary steroids day 2 (data not shown).

**TABLE 2:** Estimated sweat production for controls and athletes’ day 1 (non-training period) and day 2 (training period).

Parameter	Controls	Athletes’ day 1	Athletes’ day 2
n	26	30	30
Sweat production (L)	0.3 (−0.31–1.27)	0.3 (−0.55–1.00)	0.73 ± 0.90 *

Values presented as mean ± SD, or median and interquartile range (25th-75th percentile) depending on distribution.

For comparison between athletes’ day 1 and day 2 the Wilcoxon matched pair test was performed and for comparison between all three groups the Kruskal–Wallis test was applied.

L = liters.

*< 0.05 as compared to athletes’ day 1.

### 3.3 Steroids excreted in urine

**FIGURE 2:**
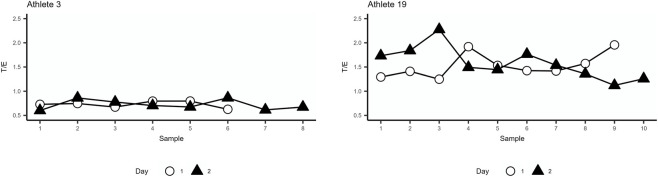
The urinary Testosterone/Epitestosterone (T/E) ratio for participants 3 and 19 showing typical concentration pattern over the two study days. X-axis spacing does not reflect actual time intervals, only chronology. See sample collection scheme in Figure 1 for details.

The intra-individual variation of the urinary steroid profile and ratios were calculated using the spot urine samples, see Figure 2 for examples of two athlete collections. There was no statistical difference in how many spot urine samples were collected from each group (median of controls 6.0 (5.0-8.0) and median of athletes’ day 1: 7.0 (6.0-7.0) and day 2: 7.0 (5.0-8.0)). No significant difference in CV % was observed when comparing controls with athlete (day 1) or between both athlete days. The relative standard deviation of the subjects can be found in Table 3.

**TABLE 3:** Relative standard deviation (CV%) of the urinary ABP steroids in controls and athletes.

Parameter	Controls	Athletes, day 1	Athletes, day 2
n	26	30	30
Androsterone	34.1 (4.2–63.7)	32.5 (10.0–74.6)	35.7 (11.7–98.9)
Etiocholanolone	36.0 (4.7–74.0)	34.8 (12.8–79.3)	36.0 (18.7–96.7)
Epitestosterone	34.7 (10.6–89.1)	31.7 (17.1–95.9)	35.6 (9.6–90.5)
Testosterone	40.2 (15.1–80.6)	33.7 (15.2–94.7)	36.8 (12.4–115.8)
5α-androstanediol	31.0 (11.6–60.2)	26.1 (7.5–76.7)	31.4 (14.4–100.6)
5β-androstanediol	36.1 (12.0–69.1)	36.2 (14.1–82.3)	36.2 (16.4–121.0)
A/Etio	10.9 (5.5–28.4)	11.1 (3.7–17.3)	10.7 (5.2–17.6)
T/E	13.0 (3.2–47.2)	14.9 (6.7–87.8)	16.2 (4.9–51.6)
5αAdiol/E	15.7 (1.3–65.1)	19.2 (5.0–50.2)	16.1 (9.2–57.1)
5αAdiol/5βAdiol	12.0 (6.3–29.3)	12.7 (5.0–48.5)	13.1 (3.8–34.5)
A/T	17.4 (8.4–28.5)	15.4 (9.0–87.6)	15.4 (4.1–47.9)

Values are presented as median and interquartile range (25th-75th percentile).

5αAdiol = 5α-androstanediol, 5βAdiol = 5β-androstanediol, A = androsterone, E = epitestosterone, Etio = Etiocholanolone, n = number, T = testosterone.

No significant differences were observed.

The total levels of ABP urinary steroids excreted for 24 h were compared between sedentary controls and athletes’ (day 1 and day 2), see Table 4. Significant differences were observed in the concentrations of A and 5αAdiol where the sedentary controls excreted significantly higher levels than the athlete’s day 2 (p = 0.04 and p = 0.03, respectively) (Figure 2). For the remaining urinary metabolites, there were no significant differences between the groups. Moreover, no significant difference in the 24 h excretion levels of the ABP metabolites between day 1 and day 2 of the athletes could be discerned.

**TABLE 4:** Urinary ABP steroids in controls and athletes.

Parameter	Controls	Athletes, day 1	Athletes, day 2
n	26	30	30
Testosterone	5.36 (2.86–8.56)	3.95 (2.98–8.00)	4.27 (2.66–6.85)
Epitestosterone	5.02 (3.69–14.7)	6.10 (2.66–7.85)	6.13 (2.86–9.41)
Androsterone	1906 (1411–3035)	1426 (1048–1969)	1415 (1099-1843)*
Etiocholanolone	1412 (1200–2309)	1791 ± 991	1843 ± 862
5α-androstanediol	22.31 (14.24–34.31)	14.41 (10.58-20.83)*	14.28(11.32-21.32)*
5β-androstanediol	36.07 (23.67–83.08)	54.61 (22.37–111)	57.08 (25.75–101)

Values presented as median and interquartile range (25th-75th percentile) and median ±SD, when normally distributed.

For comparison of Etio between athletes’ day 1 and day 2 a paired T-test was used, for all remaining urinary ABP, steroids the Wilcoxon matched pair test was performed and for comparison between all three groups the Kruskal–Wallis test was applied followed by Dunn´s multiple comparison test was performed.

ABC, athlete biological passport, n = number.

*< 0.05 as compared to controls.

**FIGURE 3:**
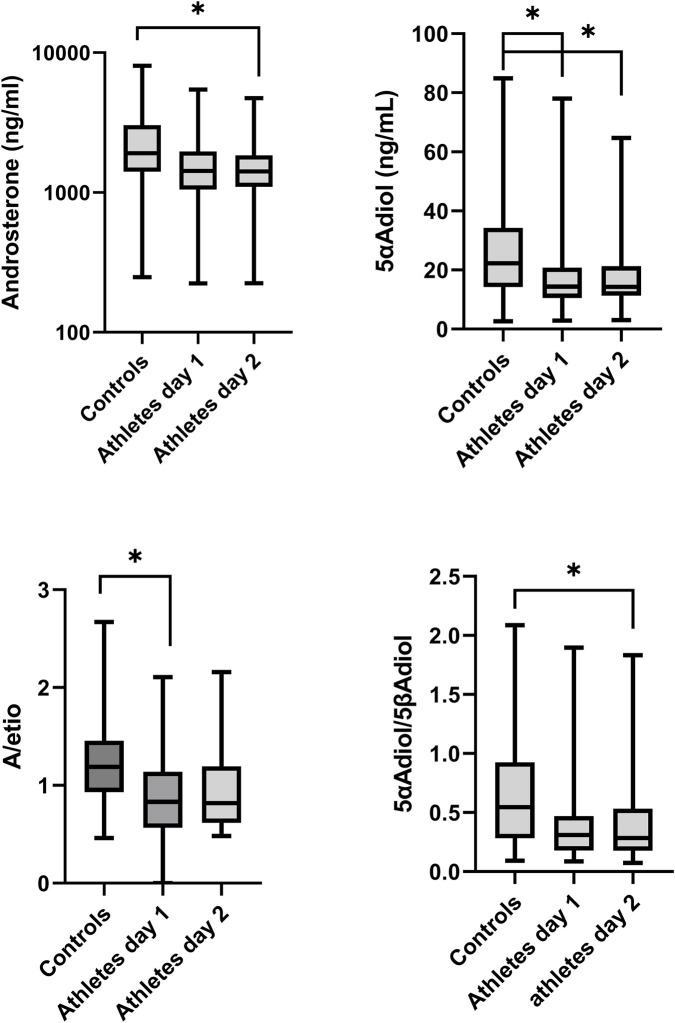
Boxplots of urinary metabolite androsterone (A) and 5αAdiol (5α-androstanediol) concentrations and A/Etiocholanolone (Etio) and 5αAdiol/5β-androstanediol (5βAdiol) ratios in sedentary controls and athletes’ day 1 and day 2 showing significant differences. All concentrations are adjusted to SG.

The ABP ratios between sedentary controls and athletes were compared. A significantly lower 5αAdiol/5βAdiol ratio was found in the athlete’s day 2 (p = 0.02) and significantly lower A/Etio ratio in the athlete’s day 1 (p = 0.03, respectively) (Figure 3) compared to the controls.

### 3.4 T and A4 in DBS

Four research participants failed to collect one of their DBS-home samples resulting in three missing DBS sample 3 (post-training sample) and one missing DBS sample 2 (pre-training sample).

**FIGURE 4:**
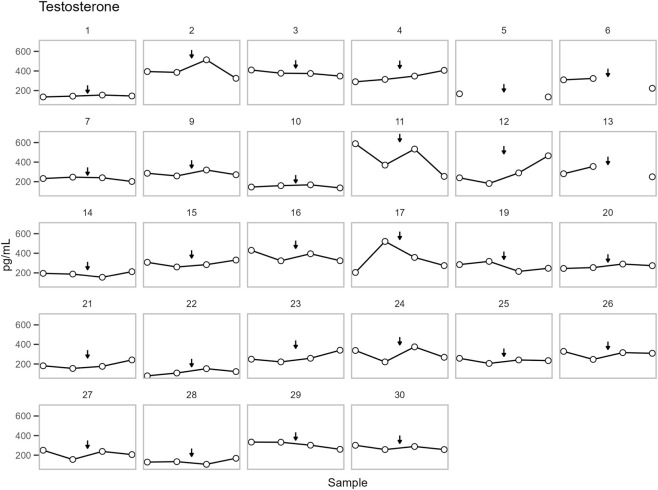
The concentration of testosterone in DBS for each participant. Points are shown in chronological order, but X-axis spacing does not reflect actual time intervals. Training is indicated by an arrow. See sample collection scheme in Figure 1 for details. The concentrations are presented in pg/mL, multiply with 0.00347 for nmol/L.

**FIGURE 5:**
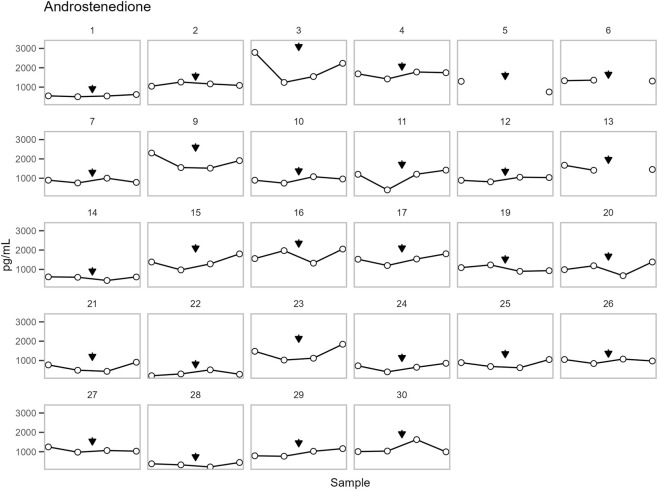
The concentration of androstenedione in DBS for each participant. Points are shown in chronological order, but X-axis spacing does not reflect actual time intervals. Training is indicated by an arrow. See sample collection scheme in Figure 1 for details. The concentrations are presented in pg/mL, multiply with 0.00349 for nmol/L.

**FIGURE 6:**
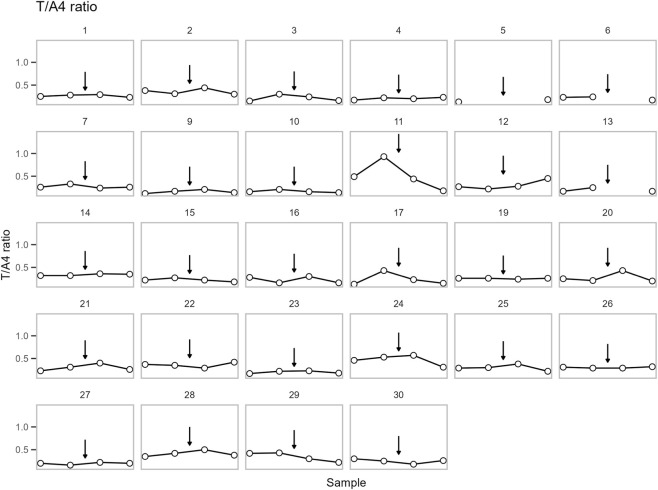
The concentration of the Testosterone/androstenedione (T/A4) ratio in DBS for each participant. Points are shown in chronological order, but X-axis spacing does not reflect actual time intervals. Training is indicated by an arrow. See sample collection scheme in Figure 1 for details.

All parameters have been plotted independently for each participant in Figures 4–6. The excluded participants 8 and 18 each presented a single outlier, with T concentrations: 207 (0.72), 5315 (18.44), 248 (0.86), 239 (0.83) pg/mL (nmol/L) and 227 (0.79), 212 (0.74), 3322 (11.53), 203 (0.70) pg/mL (nmol/L), respectively.

**FIGURE 7:**
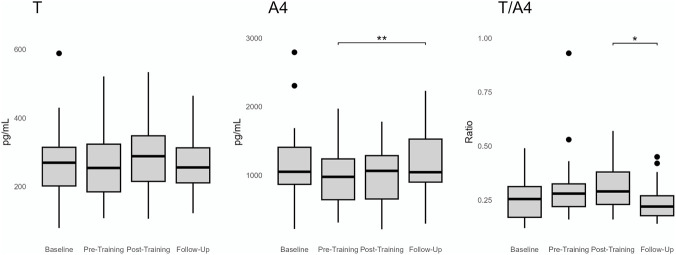
Group statistics of the dried blood spot samples over the four sampling points (Baseline, pre-training, post-training, follow-up). Significance level represents the Bonferroni corrected post-hoc analysis (paired T-test) *< 0.05, **<0.01.

No significant change in T was observed across samples. On the other hand, a significant change was observed for A4 and the T/A4 ratio (p = 0.0004 and p = 0.007, respectively). Post-hoc analysis found no significant change in A4 between pre- (DBS sample 2) and post-training (DBS sample 3). However, A4 increased from pre-training (DBS sample 2) to follow-up (DBS sample 4) (p = 0.002). Whereas T/A4 decreased from post-training (DBS sample 3) to follow-up (DBS sample 4) (p = 0.033) (Figure 7).

## 4 Discussion

To our knowledge this is the first study investigating the urinary steroid profile in 48 and 24 h urine collection, in female athletes compared to untrained controls and in the athletes between a training day and a non-training day. Our hypothesis was that the athletes would eliminate less urinary steroids than the controls in agreement with results from our previous study (Eklund et al., 2021). However, the total amount of steroids excreted in urine showed only significantly lower concentrations of A and 5αAdiol in the athletes. The reason why only 5α metabolites showed a difference is not known. It could be a random finding, or it may be that A is the predominant metabolite excreted via sweat (Thieme et al., 2003), and subsequently primarily affected. As expected, the estimated sweat production was higher during the training day (day 2) compared to day 1 among the athletes. There was, however, a large inter-individual variation in the estimated perspiration, which could be due to the various training activities the participants were engaged in. Moreover, the total sweat production calculation may be limited when considering an entire day, especially considering the estimation error of sweat loss in cooler environments mentioned by Cheuvront et al. (2002).

The reason for not seeing similar differences on the urinary steroid profile between female athletes and controls as in Eklund et al. (2021), despite power analyses, could be trait differences in the study participants. In our previous study, female Olympic athletes were included, i.e., individuals training at an extreme level with the goal of competing in upcoming Olympic Games. Even though the inclusion criteria for the athletes in this study were to exercise 2–3 h per day, there was no criteria for competing or the intensity of the training nor information regarding how well-trained the study subjects were. Subsequently, the Olympic athletes included in our previous study were likely more well-trained than the athletes included in the present study. Therefore, there is a possibility that there was not a high enough training difference between the groups in this study to observe a difference in all urinary steroids.

There was no clear acute effect of training on the urinary levels. As the urine samples were not taken at specific time points after the training session (varied from collected just after finishing the training session to up to 2 h and 15 min after) it was not possible to assess an acute effect of the urinary steroids. Other training studies have shown a direct decrease in urinary steroids that normalizes within hours (Timon Andrada et al., 2007; Timon et al., 2008b; Maynar et al., 2010).

The acute effect on circulatory T and A4 on the other hand could be evaluated as all participants collected DBS directly before and after their training session. The lack of increase in serum T further supports the notion that training intensity might not have been high enough. It has been shown that the training-intensity threshold for an androgen increase in serum may depend on fitness level. It has been reported that non-athletes significantly responded as early as 40% maximal oxygen consumption (VO_2_ max), whereas athletes did not respond below 90% maximal effort (Sato et al., 2016).

In women, serum T has been shown to increase directly after acute exercise and thereafter returning to resting values or lower within hours (Enea et al., 2011; Hackney and Willett, 2020). Whereas effects of long-term training on serum androgens have shown both elevated and reduces levels (Enea et al., 2011; Swain et al., 2022), these conflicting results are likely due to variations in study design. Also, training load with inadequate recovery time can influence the hormonal response to exercise. The strength of our study design is that the participants performed their training sessions after 2 days recovery period.

We found that T in DBS remained stable throughout the study, whereas significant changes were observed for A4 and the T/A4 ratio. However, these changes were not seen directly between pre-training (DBS sample 2) and post-training (DBS sample 3). Instead, the changes in A4 and the T/A4 ratio were observed between pre-training and follow-up and post-training and follow-up, respectively. The follow-up DBS samples were collected in the morning the day after the study participants performed their regular training regime. The pre-and post-training samples were collected at different time-points during the day (differing from in the forenoon to late evening) depending on when the study participant performed their regular exercise routine.

As the sample time during the day differs between the follow-up samples and the pre- and post-training samples, diurnal effects on A4 concentrations may explain our results. Both A4 and T are known to be higher in morning samples compared to samples taken later during the day (Burger, 2002; Davison and Bell, 2006). The stability of T, in contrast to the variability observed in A4 between DBS time points, may speculatively be explained by differences in diurnal variation.

Unfortunately, serum A4 has not been investigated to the same extent as serum T in relation to exercise in women. Further studies are needed to determine how serum ABP markers are influenced by intensive exercise in female athletes both acute and more long-term effects. In addition, physical activity performed under stress (e.g., competition vs. training) may also affect steroid disposition (Mullen et al., 2020), complicating the design of human exercise studies.

The T and A4 DBS concentrations were in same range as noted in previous DBS studies conducted in healthy young women (Salamin et al., 2022b; Desai et al., 2024), except two DBS samples, where surprisingly, T in male ranges was quantified. From the T administration study, Salamin et al. (2022b) concluded that high capillary testosterone concentration in DBS is highly likely to reflect a direct contact with exogenous testosterone gel. In our study, no T was administered, however it may be possible that the participants encounter T gel from a T-using partner, gym equipment, etc., resulting in T residues on the skin. Both the measured supra-physiological T were from DBS collected at home, and the T levels were normal in the adjacent samples, further supporting an exogenous contamination rather than a pathological explanation.

A limitation with the study design is the heterogenicity in regard to type of training as the participants did their regular- and not a standardized training session. Furthermore, for the urinary- and DBS sampling we could not consider the phase of the menstrual cycle. During the ovulation phase of a normal menstrual cycle there is a small increase in serum A4 as well as T lasting for about 24–48 h (Handelsman et al., 2018; Knutsson et al., 2023). Furthermore, urinary androgens, especially E, fluctuate during the menstrual cycle with a mid-cycle peak (Schulze et al., 2021). However, considering that the blood variation in T and A4, seen in some participants, was not consistent, as well as the sample collection of T and A4 in DBS being within 24 h for all four samples and within 2–3 h for sample 2 and 3, we find it unlikely that the hormonal variations in blood could be explained by menstrual cycle phase. However, as the urinary androgens were collected on 2 consecutive days, menstrual cycle phase could potentially have influenced the comparison of urinary androgens between athletes and controls. Additionally, HC use was allowed. Even so, the frequency of HC use was comparable between groups. Since doping tests are collected randomly, we believe that results presented here are relevant in an anti-doping context. Regarding sweat volume calculations, because sample collection spanned more than a year, environmental variation may have influenced fluid loss; however, this impact is reduced through intraindividual comparisons and paired statistical analyses.

## 5 Conclusion

We found that the urine steroids A and 5αAdiol were lower in the athletes than sedentary controls. Furthermore, in the athletes, T in DBS was not affected by training. No change was observed in A4 directly before and after training although a slight increase was observed between pre-training and the sample taken in the morning the day after the training session. The T/A4 ratio slightly decreased between directly post-training and the day after the training.

These findings suggest that T, A4 and the T/A4 ratio are not acutely affected by training. As the sample time during the day differed for the pre-, post-training and follow-up samples these minor changes in A4 and the T/A4 ratio may be due to diurnal variations and not training dependent effects. These results may be of interest when interpreting results of the ABP.

## Data Availability

The raw data supporting the conclusions of this article will be made available by the authors, without undue reservation.
